# Evaluation of Adjuvant Chemotherapy-Associated Steatosis (CAS) in Colorectal Cancer

**DOI:** 10.3390/curroncol28040265

**Published:** 2021-08-09

**Authors:** Michelle C. M. Lee, Jacob J. Kachura, Paraskevi A. Vlachou, Raissa Dzulynsky, Amy Di Tomaso, Haider Samawi, Nancy Baxter, Christine Brezden-Masley

**Affiliations:** 1 St. Michael’s Hospital, 30 Bond St, Toronto, ON M5B 1W8, Canada; mcm.lee@mail.utoronto.ca (M.C.M.L.); Paraskevi.Vlachou@unityhealth.to (P.A.V.); rdzulyns@uwo.ca (R.D.); Haider.Samawi@unityhealth.to (H.S.); Nancy.Baxter@unityhealth.to (N.B.); 2 Medical Sciences Building, 1 King’s College Circle, University of Toronto, Toronto, ON M5S 1A8, Canada; 3 Mount Sinai Hospital, 1284-600 University Avenue, Toronto, ON M5G 1X5, Canada; Jkachura@bu.edu (J.J.K.); amy.ditomaso@sinaihealth.ca (A.D.T.); 4 Lunenfeld-Tanenbaum Research Institute, 600 University Ave, Toronto, ON M5G 1X5, Canada

**Keywords:** fatty liver, chemotherapy, colorectal neoplasm

## Abstract

Chemotherapy-associated steatosis is poorly understood in the context of colorectal cancer. In this study, Stage II–III colorectal cancer patients were retrospectively selected to evaluate the frequency of chemotherapy-associated steatosis and to determine whether patients on statins throughout adjuvant chemotherapy develop chemotherapy-associated steatosis at a lower frequency. Baseline and incident steatosis for up to one year from chemotherapy start date was assessed based on radiology. Of 269 patients, 76 (28.3%) had steatosis at baseline. Of the remaining 193 cases, patients receiving adjuvant chemotherapy (*n* = 135) had 1.57 (95% confidence interval [CI], 0.89 to 2.79) times the adjusted risk of developing steatosis compared to patients not receiving chemotherapy (*n* = 58). Among patients who underwent chemotherapy, those using statins for pre-existing hyperlipidemia (*n* = 37) had 0.71 (95% CI, 0.10 to 2.75) times the risk of developing steatosis compared to patients who were not prevalent users of statins (*n* = 98). Chemotherapeutic treatment of Stage II–III colorectal cancer appears to be consistent with a moderately increased risk of steatosis, although larger studies are necessary to assess the significance of this observation. Prospective trials should be considered to further explore the potential for protective use of statins in this curative patient population.

## 1. Introduction

Hepatic steatosis is characterized by the infiltration and accumulation of triglyceride within the liver parenchyma [1]. Steatosis is the most common liver disease in Canada, affecting approximately 20% of Canadians without obvious risk factors such as alcohol abuse [2]. Progression of simple steatosis results in steatohepatitis, in which inflammation, hepatocyte damage and fibrosis ensue. More ominous complications of the spectrum of fatty liver diseases include cirrhosis and hepatocellular carcinoma [3,4]. While fatty liver is commonly associated with excessive alcohol consumption, hepatic steatosis has various other etiologies that implicate a decreased capacity of the liver to regulate lipid storage and mobilization within the body. Metabolic syndrome, a collection of conditions including abdominal obesity, insulin resistance, hypertension and hyperlipidemia, is the strongest risk factor for non-alcoholic fatty liver disease (NAFLD) [5]. Steatosis has also been attributed to hepatotoxicity induced by drugs, such as anti-tumour agents [6]. Fatty liver resulting from chemotherapy is known as chemotherapy-induced acute steatohepatitis (CASH) [7]. Both metabolic syndrome- and drug-induced fatty liver are thought to result from a pathological decrease in beta-oxidation due to the toxic accumulation of reactive oxygen species (ROS) in hepatocytes [5,7].

There is currently no approved pharmacologic treatment for hepatic steatosis, whether metabolic syndrome- or drug-induced. Among various candidate drugs under investigation, statins have been considered as a potential therapeutic option, and were recently shown to effectively reduce the risk of NAFLD development in a large population-based study [8].

Colorectal cancer (CRC) consistently ranks as one of the leading causes of cancer-related deaths in Canada [9]. The current standard guidelines for adjuvant chemotherapy in stage II and III CRC consists of the combined use of fluorouracil and oxaliplatin (FOLFOX), capecitabine and oxaliplatin (XELOX) or fluoropyridine alone [10,11,12]. The use of 5-fluorouracil (5-FU) has previously been associated with the development of steatosis in CRC patients [13,14]. However, the effects of the current chemotherapy regimen on the incidence of steatosis in the curative CRC patient population remain largely unexplored.

Medical oncologists at St. Michael’s Hospital in Toronto, Canada, anecdotally observed that CRC patients receiving adjuvant chemotherapy appeared to develop fatty liver at a higher rate than expected when seen in follow-up, based on imaging. In the absence of studies exploring the role of statin therapy in chemotherapy-associated steatosis (CAS), we speculated that the protective benefit of statins in fatty liver at large may translate to prevention of CAS among patients who are receiving statins at the time of their chemotherapy.

Thus, in this study, we sought to describe the frequency of CAS in stage II–III CRC patients and to determine the incidence of CAS in patients who were prevalent users of statins during their adjuvant chemotherapy.

## 2. Materials and Methods

### 2.1. Patients and Data Collection

We investigated the potential association between adjuvant chemotherapy and the development of steatosis among patients with stage II–III CRC. Following initial conception of the study, the authors retrospectively identified patients who were treated and followed up by a medical oncologist at St. Michael’s Hospital, Toronto, Canada, between 1 January 2006 and 1 January 2017. The electronic medical records of these patients were reviewed. Patient characteristics including sex, age at diagnosis, and Body Mass Index (BMI), as well as baseline comorbidities including type 2 diabetes mellitus, hyperlipidemia, and hypertension, were collected. Other variables included clinical data pertaining to their cancer and variables that may influence steatosis development, such as tumour location, whether primary surgical resection was performed, pelvic radiation status, steroid use, statin use, alcohol consumption, and duration and type of adjuvant chemotherapy received. This study was approved by the St. Michael’s Hospital Research Ethics Board (approval number: 18-166).

### 2.2. Determination of CAS Status

CAS was determined through a review of radiology reports, and images were reviewed by a single radiologist to maximize inter-rater reliability. Imaging modalities included contrast-enhanced abdominal CT, abdominal ultrasound, and liver or abdominal MRI. Areas of focal fatty sparing of the liver adjacent to the gallbladder and porta hepatis, absolute value of liver density less than 40 HU or a density difference greater than 25 HU between the spleen and liver on contrast-enhanced CT, increased echogenicity of the liver, attenuation of the ultrasound wave, loss of definition of the diaphragm, and poor delineation of the intrahepatic architecture on ultrasound and signal drop of liver parenchyma on the T1 weighted out of phase imaging on MRI was considered fatty liver [15,16,17,18]. Positive findings on any of the modalities qualified as presence of fatty liver. Designation of fatty liver status was made regardless of the pattern or geography of lipid distribution. Liver biochemical and function tests were not recorded and thus could not be considered in the analysis.

### 2.3. Statistical Analysis

A log binomial regression model was used to calculate adjusted relative risks. Variables found to be associated with both the exposure and outcome and thus, probably confounders, were selected as covariates based on a review of relevant literature. Directed acyclic graphs were used to identify a minimally sufficient set of covariates to control potential confounding in the final adjusted model (Supplementary Figures S1 and S2). Covariates included sex, BMI, type 2 diabetes mellitus status, hyperlipidemia status, and steroid use in the analysis of the association between adjuvant chemotherapy exposure and steatosis outcome, while sex, BMI, type 2 diabetes mellitus status and hyperlipidemia status were adjusted for in the analysis of the association between statin use and steatosis outcome [5,19,20,21,22]. All statistical analyses were performed using SAS 9.4 (SAS Institute, Cary, NC, USA).

## 3. Results

**Figure 1:**
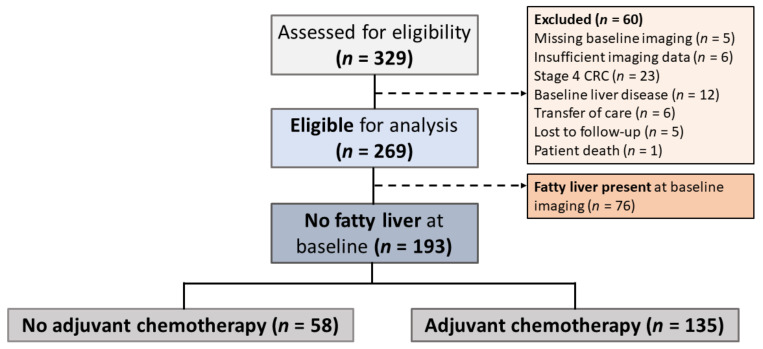
Study Participant Selection. Stage II–III colorectal cancer (CRC) patients were screened for eligibility prior to data collection, and those in whom fatty liver was present at baseline were excluded from further analyses. The 187 patients who did not have signs of hepatic steatosis at baseline were further divided into two groups based on their receipt of adjuvant chemotherapy.

Overall, 329 patients who were diagnosed with stage II–III CRC at St. Michael’s Hospital from 1 January 2006 to 1 January 2017 were assessed for eligibility. Out of the 269 patients deemed eligible for analysis, 76 (28.3%) had fatty liver at baseline imaging, prior to treatment with adjuvant chemotherapy. The remaining 193 patients who did not have fatty liver at baseline were further divided by whether they received curative chemotherapy (Figure 1).

The characteristics of the two patient groups are outlined in Table 1. There was a greater proportion of males among patients who received adjuvant chemotherapy (81/135, 60.0%) compared to the no adjuvant chemotherapy group (24/58, 41.4%). The mean age of diagnosis was higher in the no adjuvant chemotherapy group (68.0 ± 14.1 vs. 59.9 ± 11.4). The proportion of patients whose BMI was ≥ 25 was comparable between the two groups. A higher proportion of individuals who did not receive chemotherapy had other risk factors for NAFLD present, including type 2 diabetes mellitus (19.0% vs. 13.3%), hypertension (51.7% vs. 38.5%), and hyperlipidemia (43.1% vs. 28.9%); therefore, statin use was also higher in this group (36.2% vs. 27.4%). A small number of patients in each group was using steroids, with a slightly greater proportion seen in the no adjuvant chemotherapy group (13.8% vs. 6.7%). More individuals in the adjuvant chemotherapy group received pelvic radiation (10.3% vs. 38.5%). Both groups exhibited similar rates of reported alcohol use.

We found that 52 of 135 patients (38.5%) who received adjuvant chemotherapy developed steatosis within one year of follow-up, compared to 14 of 58 patients (24.1%) who did not receive chemotherapy (Relative Risk [RR] 1.57, 95% confidence interval [CI] 0.89 to 2.79) after adjustment for sex, BMI, type 2 diabetes mellitus, hyperlipidemia, and steroid use. The point estimate is consistent with moderately increased risk of steatosis, but with wide confidence intervals (Table 2).

We then examined the 135 patients who received adjuvant chemotherapy. Of these patients, 103 individuals were treated with an oxaliplatin-containing regimen, which is FOLFOX. In our study cohort, we did not observe a significant difference in the risk of developing steatosis when comparing patients receiving oxaliplatin-containing chemotherapy compared to those on a 5-FU based regimen (RR 0.64, 95% CI 0.30 to 1.38) after adjusting for sex, BMI, type 2 diabetes mellitus, hyperlipidemia, and steroid use (Table 3). A comparison of the effects of oral capecitabine versus intravenous 5-FU (Table 4) did not suggest the existence of a significant association between route of chemotherapy administration and risk of steatosis in the same group of patients (RR 1.56, 95% CI 0.65 to 3.73).

The demographics of 135 patients who were treated with chemotherapy were summarized based on statin administration status, shown in Table 5. Patients were considered to have positive statin administration if they were found to be prevalent users of a statin at the time of chemotherapy initiation. There was a greater proportion of males in the group of patients on statin therapy (70.3% vs. 56.1%). This group also exhibited a mildly higher proportion of overweight or obese individuals compared to the no statin group (54.0% vs. 43.9%). Notably, all patients who were administered statins had pre-existing hyperlipidemia, and a higher proportion of patients in this group had hypertension (73.0% vs. 25.5%).

Among patients who were on statins at the time of their adjuvant chemotherapy, 11 of 37 (29.7%) patients developed steatosis within one year; however, 41 of 98 (41.8%) patients who did not receive statin treatment developed steatosis. The relative risk of developing steatosis was not significantly different regardless of statin therapy status at the time of adjuvant chemotherapy (RR 0.45, 95% CI 0.10 to 2.75) after adjusting for sex, BMI, type 2 diabetes mellitus, and hyperlipidemia (Table 6).

## 4. Discussion

In the present study, the adjusted relative risk of adjuvant chemotherapy reflected a moderately increased risk of steatosis, although the confidence intervals were wide. According to existing literature, the frequency of hepatic steatosis development in CRC patients receiving 5-FU ranges from 30–47% [14,23,24]. Miyake et al. also retrospectively reported that 34.9% of CRC patients treated with 5-FU developed hepatic steatosis, a rate significantly higher than that seen in the no treatment group [13]. Some researchers have suggested that hepatic steatosis induced by 5-FU-containing therapy is reversible, although treatment regimens used in these studies consisted of additional agents such as levamisole and interferon alpha-2a [23,24]. In contrast, Vigano and colleagues examined the impact of oxaliplatin- and irinotecan-based chemotherapy in metastatic CRC patients and reported that while other types of chemotherapy-induced liver injury such as sinusoidal dilatation and nodular regenerative hyperplasia regressed in the majority of cases, steatosis and steatohepatitis persisted [25]. Thus, the extent of chemotherapy-associated steatosis and steatohepatitis reversibility and the implications of these findings on patients treated using the current Canadian chemotherapy guidelines need to be further elucidated.

The use of oxaliplatin as part of the chemotherapy regimen did not appear to affect the risk of steatosis in our analysis, barring perhaps a non-significant, mildly lower rate of steatosis development in those receiving oxaliplatin compared to those on treatment regimens not containing oxaliplatin. Despite its frequent association with liver injury, oxaliplatin is classically known to damage the liver via sinusoidal dilation and is thought to have a limited role in inducing steatohepatitis [26]. However, there has yet to be a clear consensus regarding the effect of oxaliplatin on steatosis, especially in light of findings such as that of Lu and colleagues, who showed that oxaliplatin aggravates existing steatosis in a non-alcoholic fatty liver disease mouse model [27]. More widely accepted is the association of CASH with irinotecan-based chemotherapies, which were not explored in the present study but would be highly pertinent for future investigations [28].

Since the FDA approval of capecitabine in 2001 as a chemotherapeutic agent, the oral route of 5-FU delivery became a mainstay of colorectal cancer treatment, alongside intravenous infusion. While liver enzymes play a central role in the three-step enzymatic cascade of capecitabine activation to its active metabolite, hepatotoxicity is considered a relatively rare side effect of capecitabine due to its selective activation within the tumor tissue [29,30]. There are however reports of capecitabine-induced liver injury, including cases of acute liver injury and hepatic steatosis [31,32]. In our study, the adjusted risk of steatosis development was 1.56 when compared with intravenous regimens of 5-FU administration, but this point estimate was not statistically significant.

Considering the low survival rates of CRC patients in the absence of treatment, adverse effects of CRC chemotherapy have been regarded as inevitable consequences of a necessary life-saving measure. Chemotherapy-associated steatosis is pathologically indistinguishable from NAFLD, which has a benign onset as simple hepatic steatosis, but can asymptomatically progress to steatohepatitis [7]. NAFLD is associated with an increased risk of cirrhosis, and hepatocellular carcinomas that arise in NAFLD patients are associated with a higher morbidity than those with other etiologies [3,33].

Despite these risks, current treatment for hepatic steatosis is limited to changes in lifestyle to mitigate cardiovascular risk factors [34]. The development of pharmacological therapeutic agents is in progress, with promising candidates including obeticholic acid, firsocostat, and elafibranor [35]. Statins have been implicated as a potential therapeutic agent for steatosis, but their efficacy has been somewhat inconclusive according to existing literature. Statins competitively inhibit HMG-CoA reductase, a key enzyme in cholesterol biosynthesis, to exert potent LDL-cholesterol lowering effects. Hyperlipidemia is highly prevalent in the population commonly affected by NAFLD, thus patients with hepatic steatosis tend to be aggressively treated using statins [36,37,38]. There have been indications of histologically determined benefit from statin use in animal models of NASH, as well as post hoc analyses of randomized controlled trials, which revealed that atorvastatin may alleviate NAFLD [39]. Other studies showed a lack of a statistically significant difference between statin users and the control group in this regard, highlighting the need for randomized controlled trials [40,41]. Nevertheless, the recent population-based study by Lee and colleagues demonstrated a significant reduction in the development of NAFLD in patients who received statin therapy, supporting the potential for statins to confer protective benefits in preventing steatosis [8]. In our study, all patients receiving statins had pre-existing dyslipidemia. We have shown that patients who were administered statins at the time of their chemotherapy may be at a lower risk of developing CAS than those treated with chemotherapy alone, although larger studies are necessary to validate the statistical significance of these results. To our knowledge, this is the first study to examine the potential protective benefit of statins in the context of hepatic steatosis driven by chemotherapy. Given that hyperlipidemia is strongly associated with hepatic steatosis, it is possible that the effect of statin treatment was underestimated in our patient cohort.

In this study, the primary mode of determining steatosis status in patients included a review of the medical records and the abdominal images (CT, ultrasound and MRI) by a single radiologist. MRI exhibits the highest sensitivity for detecting hepatic lipid infiltration and can detect as little as 5% steatosis in the liver at a sensitivity of 76.7–90.0% and a specificity of 87.1–91% [42]. Ultrasound can detect ≥20% steatosis with a sensitivity of 79.7% and specificity of 86.2% [42]. Contrast-enhanced CT has a detection rate comparable to that of ultrasound; its sensitivity ranges from 54% to 93% and specificity from 87% to 93% [42]. While some patients underwent ultrasound and MRI in addition to CT, since the primary goal of follow-up imaging was to determine whether the patient developed cancer metastasis, contrast-enhanced CT was the most regularly performed imaging modality among the largest number of patients in our study. Since not every patient’s steatosis status was verified using a second imaging modality in addition to a CT, there may have been inconsistencies in the reported steatosis status; this is a major limitation of the present study. However, there have been other studies that used contrast-enhanced CT as the sole imaging modality to assess hepatic steatosis [43].

Another limitation of this study is the relatively small sample size, particularly for the cohort of patients receiving statins. The sample size of 37 in the statin group meant that the power of the statistical analysis was smaller than the widely accepted threshold of 80%. This study was also limited in that many potentially useful clinical and demographic data such as duration of statin administration and lifestyle factors contributory to steatosis could not be collected due to the retrospective nature of the investigation. Other data that may have further enriched our findings include trending of lipid profiles as well as liver biochemical and function tests. However, while elevated levels of liver biochemical tests often correlate with a diagnosis of fatty liver, a high proportion of patients with NAFLD exhibit normal liver profiles, making these tests inappropriate diagnostic markers that may not have drastically impacted the findings presented in our study [44,45]. Finally, reporting bias inherent to this retrospective study may have affected our results in the process of analyzing self-reported variables such as alcohol consumption and family history of hyperlipidemia.

## 5. Conclusions

This study sought to examine hepatic steatosis, an increasingly recognized health concern worldwide. Since drug-induced hepatotoxicity was described by Grieco et al. in 2005, there has been some research on the association between steatosis and anti-tumour drugs, although the precise impacts of CRC chemotherapy have largely been unexplored. Furthermore, there is currently a lack of medical treatment for any population affected by steatosis, regardless of etiology, although a recently published population-based study suggests that statins may confer protective benefits against the development of steatosis. Here, we observed that there is a trend towards a higher rate of CAS development within one year of follow-up among stage II–III CRC patients who received chemotherapy compared to the no treatment group. Moreover, there is evidence to believe that larger, higher power studies should be conducted to further investigate the protective benefits of statins in reducing the risk of CAS, owing to the mild reduction in the adjusted relative risk of steatosis in statin users observed in the present study. These conclusions have critical implications on the quality of life and hepatic function of patients not only in the curative setting, but may also be applicable in the setting of treatment of metastatic disease, in particular in context of patients requiring liver resections for metastases in addition to indefinite metastatic treatment which may require up to 60 cycles of 5-FU-based chemotherapy.

Based on the results of our study, it is necessary to conduct prospective studies that involve a larger cohort of patients, who are controlled for comorbidities that may confound the association between CRC, statin use and the incidence of steatosis. An assessment of the safety and efficacy of statins in a randomized controlled cohort will allow for an accurate investigation into this phenomenon that contributes to a secondary health burden for the curative CRC patient population.

## Figures and Tables

**Table 1:** Patient demographics.

	No Adjuvant Chemotherapy	Adjuvant Chemotherapy
	*n* = 58	*n* = 135
**Sex, Male**	24 (41.4%)	81 (60.0%)
**Age at diagnosis, years** (mean ± SD)	68.0 ± 14.1	59.9 ± 11.4
≤55	11 (19.0%)	50 (37.0%)
56–69	20 (34.5%)	57 (42.2%)
≥70	27 (46.6%)	28 (20.7%)
**BMI (kg/m^2^)**		
Underweight (<18.5)	0 (0.0%)	4 (3.0%)
Normal Weight (18.5–24.9)	18 (31.0%)	65 (48.2%)
Overweight (25–29.9)	19 (32.8%)	48 (35.6%)
Obese (≥30)	7 (12.1%)	15 (11.1%)
Missing	14 (24.1%)	3 (2.2%)
**Type 2 Diabetes**	11 (19.0%)	18 (13.3%)
**Hyperlipidemia**	25 (43.1%)	39 (28.9%)
**Hypertension**	30 (51.7%)	52 (38.5%)
**Tumour location**		
Right colon (ascending, transverse)	36 (62.1%)	32 (23.7%)
Left colon (descending, sigmoid)	16 (27.6%)	32 (23.7%)
Rectum	6 (10.3%)	61 (45.2%)
Multiple locations	0 (0.0%)	10 (7.4%)
**Primary surgical resection**	55 (94.8%)	134 (99.3%)
**Pelvic radiation**	6 (10.3%)	52 (38.5%)
**Steroid use**	8 (13.8%)	9 (6.7%)
**Statin use**	21 (36.2%)	37 (27.4%)
Atorvastatin	13 (61.9%)	19 (55.9%)
Pravastatin	2 (9.5%)	1 (2.9%)
Rosuvastatin	2 (9.5%)	7 (20.6%)
Simvastatin	4 (19.1%)	7 (20.6%)
**Alcohol consumption ^a^**		
None	27 (46.6%)	73 (54.1%)
Moderate	27 (46.6%)	55 (40.7%)
Frequent	4 (6.9%)	7 (5.2%)

Note: data are presented as frequency (percent), unless otherwise specified. Abbreviations: BMI (Body Mass Index), ^a^ Alcohol consumption categorization based on Canadian Centre on Substance Abuse guidelines. Moderate: <10, 15 drinks per week for females, males, respectively, Frequent: >10, 15 drinks per week for females, males, respectively.

**Table 2:** Association between adjuvant chemotherapy exposure and outcome of steatosis in patients with colorectal cancer.

	Developed Steatosis	No Steatosis	Crude Relative Risk	Adjusted ^a^ Relative Risk (95% CI)	*p*-Value
Adjuvant Chemotherapy	52 (38.5%)	83 (61.5%)	1.60	1.57 (0.89–2.79)	0.12
No Adjuvant Chemotherapy (Ref)	14 (24.1%)	44 (75.9%)	1.00	1.00	

^a^ Adjusted for sex, BMI, diabetes, hyperlipidemia, and steroid use.

**Table 3:** Oxaliplatin compared to no oxaliplatin regimens and development of steatosis.

	Developed Steatosis	No Steatosis	Crude Relative Risk	Adjusted ^a^ Relative Risk (95% CI)	*p*-Value
Oxaliplatin	36 (35.0%)	67 (65.1%)	0.70	0.64 (0.30–1.38)	0.26
Non-Oxaliplatin (Ref)	16 (50.0%)	16 (50.0%)	1.00	1.00	

^a^ Adjusted for sex, BMI, diabetes, hyperlipidemia, and steroid use.

**Table 4:** Capecitabine compared to 5-FU-containing intravenous regimens and development of steatosis.

	Developed Steatosis	No Steatosis	Crude Relative Risk	Adjusted ^a^ Relative Risk (95% CI)	*p*-Value
Capecitabine	14 (48.3%)	15 (51.7%)	1.35	1.56 (0.65–3.73)	0.32
Intravenous regimens ^b^ (Ref)	38 (35.9%)	68 (64.2%)	1.00	1.00	

^a^ Adjusted for sex, BMI, diabetes, hyperlipidemia, and steroid use. ^b^ Intravenous regimens include FOLFOX and FUFA.

**Table 5:** Statin use in patients who received adjuvant chemotherapy for their colorectal cancer.

	No Statin Administration	Statin Administration
	*n* = 98	*n* = 37
**Sex, Male**	55 (56.1%)	26 (70.3%)
**BMI (kg/m^2^)**		
Underweight (<18.5)	3 (3.1%)	1 (2.7%)
Normal Weight (18.5–24.9)	49 (50.0%)	16 (43.2%)
Overweight (25–29.9)	32 (32.7%)	16 (43.2%)
Obese (≥30)	11 (11.2%)	4 (10.8%)
Missing	3 (3.1%)	0 (0.0%)
*Comorbitities*		
**Type 2 Diabetes**	7 (7.1%)	11 (29.7%)
**Hyperlipidemia**	2 (2.0%)	37 (100.0%)
**Hypertension**	25 (25.5%)	27 (73.0%)
*Treatment*		
**Pelvic radiation**	38 (38.8%)	14 (37.8%)
**Steroid use**	7 (7.1%)	2 (5.4%)
*Adjuvant chemotherapy characteristics*		
**Duration of adjuvant chemotherapy, cycles ^a^** (mean ± SD)	8.7 ± 2.7	8.0 ± 2.8
**Type of adjuvant chemotherapy received**		
FOLFOX	84 (85.7%)	19 (51.4%)
Capecitabine	14 (14.3%)	15 (40.5%)
FUFA	0 (0.0%)	3 (8.1%)

Note: data are presented as frequency (percent), unless otherwise specified. Abbreviations: BMI, Body Mass Index; FOLFOX, oxaliplatin, fluorouracil, and folinic acid; XELODA, capecitabine; FUFA, fluorouracil and folinic acid. ^a^ Cycles of adjuvant chemotherapy are calculated based on the usual schedules of administration of FOLFOX/FUFA every 2 weeks, and capecitabine every 3 weeks.

**Table 6:** Steatosis in patients who received adjuvant chemotherapy for their colorectal cancer based on statin administration status.

	Developed Steatosis	No Steatosis	Crude Relative Risk	Adjusted ^a^ Relative Risk (95% CI)	*p*-Value
Statin use	11 (29.7%)	26 (70.3%)	0.71	0.53 (0.10–2.75)	0.45
No statin use (Ref)	41 (41.8%)	57 (58.2%)	1.00	1.00	

^a^ Adjusted for sex, BMI, diabetes, and hyperlipidemia.

## Data Availability

The data presented in this study are available on request from the corresponding author. The data are not publicly available due to patient privacy.

## Supplementary Materials

The following are available online at https://www.mdpi.com/article/10.3390/curroncol28040265/s1, Figure S1: Directed Acyclic Graph (DAG) for the potential association between adjuvant chemotherapy and development of non-alcoholic fatty liver disease (NAFLD), Figure S2: Directed Acyclic Graph (DAG) for the potential association between statin administration and development of non-alcoholic fatty liver disease (NAFLD).
