# Supplementary material for: Evaluation of Adjuvant Chemotherapy-Associated Steatosis (CAS) in Colorectal Cancer
Source: Curr Oncol. 2021 Aug 9;28(4):3030–40. doi: 10.3390/curroncol28040265 (PMC8395441; doi:10.3390/curroncol28040265)
Supplement: Supplementary file 1 [file curroncol-28-00265-s001.zip › curroncol-1261282-supplementary.pdf]

Current Oncology

Supplementary Figures

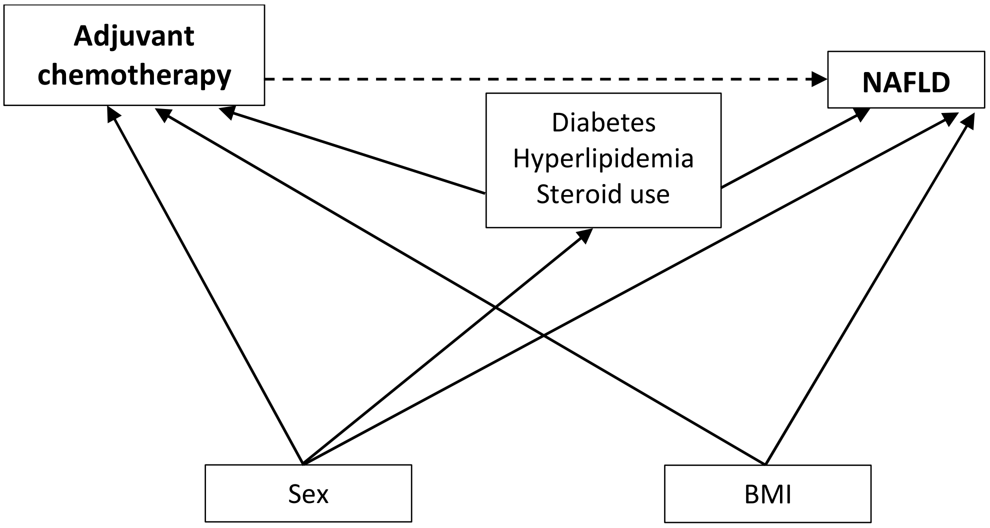

Figure S1: Directed Acyclic Graph (DAG) for the potential association between adjuvant chemotherapy and development of non-alcoholic fatty liver disease (NAFLD).

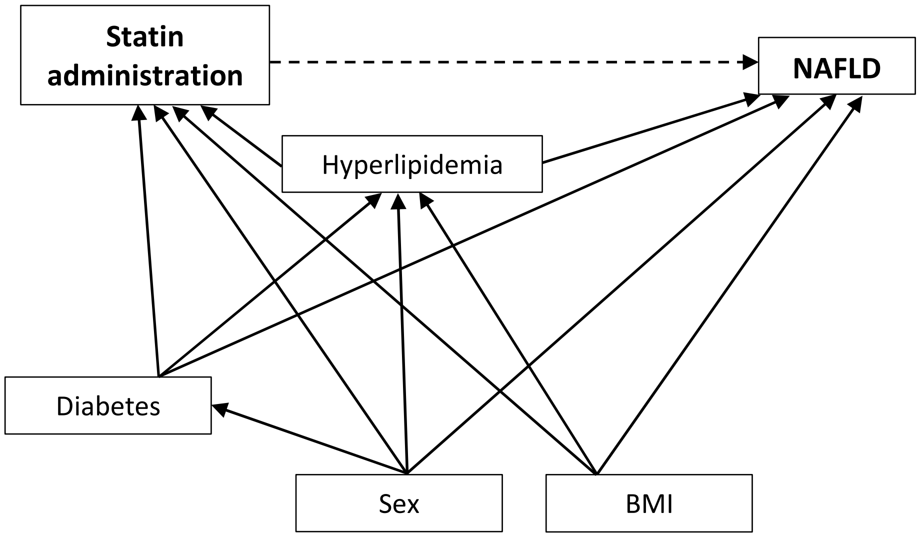

Figure S2: Directed Acyclic Graph (DAG) for the potential association between statin administration and development of non-alcoholic fatty liver disease (NAFLD).
